# Artemis: Harnessing Knowledge Graphs for Next-Generation Drug Target Prioritization

**DOI:** 10.34133/csbj.0130

**Published:** 2026-06-26

**Authors:** Vladimir Yu. Kiselev, Edward Ainscow

**Affiliations:** Alethio Therapeutics, Abingdon, Oxfordshire OX14 3NB, UK.

## Abstract

Knowledge graphs (KGs) have become an important asset in biomedical research and drug discovery by enabling the structured integration of heterogeneous biological knowledge. When combined with machine learning (ML), KGs support the identification of novel drug–target relationships, but existing approaches are often KG-centric, relying primarily on graph structure and embeddings while overlooking disease-specific biological and clinical context. Moreover, many high-impact applications depend on proprietary KG infrastructures, limiting accessibility for the broader research community. Here, we introduce Artemis, a practical and generalizable ML framework for indication-aware target prioritization that integrates public biomedical KGs with clinical evidence from the ChEMBL database. Artemis derives graph-based representations of clinically validated drug targets from multiple publicly available KGs and trains supervised ML models using disease-relevant clinical labels derived from ChEMBL. This hybrid feature space is used to train supervised ML models across 7 disease indications, with performance assessed via cross-validation and guided parameter optimization. The framework is further evaluated on emerging breast cancer targets reported at the San Antonio Breast Cancer Symposium 2024, demonstrating its ability to prioritize novel candidates. Overall, this work demonstrates that publicly available KGs can be used for actionable, translational target discovery when coupled with clinical data. Artemis provides an accessible, scalable, and cost-efficient alternative to proprietary KG platforms, thereby offering a practical solution for researchers seeking to prioritize therapeutic targets in real-world drug discovery settings.

## Introduction

Since their implementation by Google in 2012, knowledge graphs (KGs) have become a foundational technology across a wide range of research fields and applications [1]. By 2020, KGs had begun to gain traction in biomedical research, attracting substantial attention from several major pharmaceutical companies. For example, AstraZeneca developed the Biological Insights Knowledge Graph (BIKG) [2] to support internal applications such as discovery ranking of CRISPR screen hits to aid novel target discovery. Novartis employed knowledge graph embeddings (KGEs) to recommend patient selection, design, and end points for clinical trials [3]. GlaxoSmithKline applied KGs to improve detection power in small-cohort genome-wide association studies [4], and Boehringer Ingelheim proposed a novel KG construction framework [5] later used for indication expansion and drug repurposing [6].

While these examples demonstrate the versatility and value of KGs in pharmaceutical drug discovery and development, they rely on proprietary platforms, inaccessible to the broader research community. A few commercial providers—such as Clarivate (https://clarivate.com/life-sciences-healthcare/consulting-services/research-and-development-consulting/knowledge-graphs/) and Qiagen (https://digitalinsights.qiagen.com/biomedical-knowledge-base/)—offer access to curated KGs but at a considerable cost. However, in parallel, several publicly available biomedical KGs have emerged, beginning with the influential Hetionet KG [7]. AstraZeneca maintains a public GitHub repository cataloguing many of these open KGs (https://github.com/AstraZeneca/awesome-drug-discovery-knowledge-graphs/blob/main/chapters/drug_discovery_kgs.md). Community initiatives have also facilitated access and experimentation: for example, the PyKEEN framework [8] currently integrates nearly 40 standardized KGs, several of which are relevant to biomedical applications.

Recent studies on target discovery using KGs have largely focused on predicting potential drug targets either by exploiting improved graph construction and graph structural properties or by developing more advanced machine learning (ML) methods for KG representation and analysis [9–21]. While these approaches have led to methodological advances, they are fundamentally KG-centric: they treat the graph as the primary source of information and typically overlook disease-specific biological features. As a result, they do not explicitly incorporate the clinical or mechanistic context of the indication for which new targets are being sought.

More broadly, computational approaches to drug discovery have advanced across multiple fronts, including drug–target affinity prediction [22], off-target effect assessment [23], multi-omics network integration [24], drug response prediction [25], and the curation of target-centric databases [26,27]. While these methods address complementary aspects of the drug discovery pipeline, they do not perform indication-specific target prioritization from KGs—the central focus of the present work.

ChEMBL is a large, open-access, expertly curated database of chemical structures linked to quantitative bioactivity measurements against defined biological targets [28]. Its standardized assays, target annotations, and chemical representations have made it a cornerstone dataset for training and benchmarking ML models in drug discovery [29]. Despite its widespread use in ML, ChEMBL has rarely been integrated with biomedical KGs for target discovery, and systematic approaches that combine KG-derived features with clinical-stage evidence remain largely unexplored.

Here, we describe Artemis, an ML framework designed to bridge network-level biological knowledge and clinical evidence for target evaluation. Artemis leverages publicly available biomedical KGs to generate feature representations of known clinical drug targets and trains supervised ML models on indication-specific clinical labels derived from ChEMBL. This integrated feature space underpins supervised ML models trained across 7 disease indications, enabling target evaluation that reflects both biological connectivity and empirical drug–target interactions. Model performance is validated using cross-validation and parameter optimization and further tested on emerging breast cancer targets mined from abstracts from the San Antonio Breast Cancer Symposium (SABCS) 2024 [30].

This approach addresses a limitation of existing KG-centric methods by moving beyond purely structural graph signals toward indication-aware target prioritization. Artemis is particularly suited for researchers who identify candidate targets from experimental [31] or computational screens and seek to rank them using an interpretable, ML-driven framework grounded in both KG topology and clinical evidence. By relying exclusively on publicly available resources, Artemis provides a novel, practical, and accessible strategy for target evaluation with direct relevance to real-world drug discovery workflows.

## Methods

### Knowledge graphs

Every KG consists of nodes (entities, such as genes, proteins, drugs, or diseases) and edges (relationships that capture known or inferred biological interactions or associations, derived from curated databases, experimental evidence, or computational predictions). Two nodes connected by an edge create a triple. Triples are the building blocks of a KG. In our study, we focused on several publicly available biomedical KGs, all of which are included and standardized within the PyKEEN Python library [8]. Their properties are shown in Table 1.

**Table 1.** Characteristics of KGs used in the study

Name	No. of nodes	No. of relations	No. of triples	Reference
BioKG	105,524	17	2,067,997	[44]
Hetionet	45,158	12	2,250,197	[7]
OpenBioLink	180,992	28	4,559,267	[45]
PrimeKG	129,262	30	8,099,991	[46]

KGs, knowledge graphs

These KGs were selected to provide a diverse and representative set of biomedical networks varying in scale, ontology structure, and data provenance, ensuring a robust evaluation of our target discovery framework. Full details of the KGs are available in the Supplementary Notebook.

### KG embeddings

KGE is an ML technique that refers to the process of learning low-dimensional vector representations of entities and relations in a KG while preserving its structural and semantic characteristics. When KGE vectors are used as features in a downstream supervised model that predicts gene druggability, any drug–target edges retained in the graph during embedding training could encode the very label that the classifier is asked to learn, constituting data leakage. To eliminate this, we implemented a pre-embedding filtering step that removes all triples linking clinical target genes to drug or compound entities.

First, clinical target gene symbols were resolved to the identifier scheme used by each KG. For Hetionet (Gene::<entrez_id>) and OpenBioLink (NCBIGENE:<entrez_id>), the National Center for Biotechnology Information *Homo sapiens* gene_info file (https://ftp.ncbi.nlm.nih.gov/gene/DATA/GENE_INFO/Mammalia/Homo_sapiens.gene_info.gz) was used to map symbols (including synonyms) to Entrez Gene IDs. For BioKG, which indexes proteins by UniProt accession, the reviewed human proteome was queried from the UniProt Representational State Transfer (REST) application programming interface to map gene symbols to accession numbers. PrimeKG uses plain gene symbols as entity labels and was matched directly.

Drug entities were identified by KG-specific naming conventions: the “Compound::” prefix in Hetionet, the DB (DrugBank) prefix in BioKG, and the “PUBCHEM.COMPOUND:” prefix in OpenBioLink. In PrimeKG, where drug nodes carry no distinguishing prefixes, drug-related edges were instead identified by their relation type (e.g., drug_protein, drug_drug, indication, contraindication, and off-label use).

Every triple in which one argument is a clinical target gene entity and the other is a drug/compound entity (or, for PrimeKG, the relation type is drug related and involves a clinical target gene) was removed from all 3 splits (training, validation, and testing) before the splits were merged and passed to the embedding model. Aggregate filtering statistics are shown in Table 2.

**Table 2.** Statistics of the KG triples removed to prevent data leakage

Knowledge graph	Triples removed	Removed (%)
Hetionet	6,393	0.28
BioKG	17,453	0.84
OpenBioLink	135,399	2.97
PrimeKG	17,378	0.21

KG, knowledge graph

After the above filtering process to avoid data leakage, we generated embeddings of the KGs using 4 distinct KGE models: RotatE, ComplEx, DistMult, and TransE—implemented within the PyKEEN framework. For BioKG and Hetionet KGs, we adopted the best-performing hyperparameter configurations reported in a recent study [32]. These configurations have been empirically validated to produce high-quality embeddings for prediction tasks. For OpenBioLink, which was not included in the reference study, we applied the same hyperparameters as those used for Hetionet for each respective model. Similarly, for PrimeKG, we adopted the BioKG configurations for each model, with the number of training epochs reduced to 150 due to the large graph size. A fixed random seed was used across all experiments to ensure reproducibility.

### KG link predictions

Link prediction (LP) in KGs aims to infer previously unobserved relations between entities by leveraging the existing network structure and the learned embeddings of entities and relations. In addition to discovering new associations, LP can also be used to quantify the confidence or plausibility of existing relations by assigning a score to each known triple within the KG.

For each KG, we performed LP using the PyKEEN framework. Rather than predicting all possible links across the entire graph, we adopted a gene-centric approach, focusing exclusively on links between gene/protein nodes and other relevant entities, depending on the relation types defined within each KG. Because relation naming conventions vary across public KGs, we manually curated a subset of relations corresponding specifically to gene and protein associations. A detailed list and descriptions of these curated relations are provided in the Supplementary Notebook.

The output of the LP process was a gene–node matrix, where rows represent genes/proteins, columns correspond to other KG entities, and cell values denote the predicted strength of the relation between each pair. This matrix includes only the relation types already present in the original KG, ensuring that predictions remain biologically interpretable and consistent with the underlying graph schema.

### Clinical scores

To enrich the KG-derived relationships with clinical evidence, we queried the ChEMBL database (version 36) for bioactive drug molecules associated with clinical trial data and annotated therapeutic indications. All molecular variants (e.g., salts, formulations, and prodrugs) were standardized to their corresponding parent molecules, and associated information—including clinical trial phase, Medical Subject Headings (MeSH) disease annotations (https://www.nlm.nih.gov/mesh/meshhome.html), mechanisms of action, and molecular targets—was aggregated at the parent-molecule level to generate a unified dataset. Disease indications were grouped by selecting a top-level MeSH branch and including all its descendant terms. This grouping strategy was applied to a set of cancer and noncancer indications selected to reflect a combination of high disease prevalence, clinical impact, and availability of well-characterized therapeutic targets. The cancer indications represent some of the most common and intensively studied tumors, spanning distinct tissues of origin, molecular drivers, and therapeutic landscapes. The noncancer indications were included to evaluate the generalizability of the framework beyond oncology, particularly in chronic, multifactorial diseases with extensive clinical and genetic evidence bases. Together, this selection enables a systematic assessment of model performance across diverse disease areas:

• cancer indications
  ◦ breast neoplasms (breast)
  ◦ lung neoplasms (lung)
  ◦ intestinal neoplasms (bowel)
  ◦ prostatic neoplasms (prostate)
  ◦ skin neoplasms (melanoma)
• noncancer indications
  ◦ diabetes mellitus, type 2 (diabetes)
  ◦ cardiovascular diseases (cardiovascular)

To mitigate bias introduced by drugs that may act through multiple, closely related proteins, we addressed target redundancy at the gene family level. For each drug–disease combination, we identified overrepresented HUGO Gene Nomenclature Committee (HGNC) gene families [33]—defined as cases in which more than 40% of family members were targeted by a single molecule—and retained a single representative gene per family (selected alphabetically).

To represent the clinical maturity of each target, we computed a simple clinical score based on the number and clinical phase distribution of associated clinical trials (considering trials only from the last 6 years). Specifically, if a target was linked to *a* phase I trials, *b* phase II trials, and *c* phase III trials, its score was calculated as

$$\begin{array}{c} \text{Clinical score}=\;a+\;2b+\;3c \end{array}$$ (1)

Additional approval bonus (+20) was added to the clinical score if a target has at least one approved drug in each disease indication. This weighting scheme assigns higher importance to targets evaluated in more advanced clinical stages and even higher importance to the ones that are targeted by approved drugs, reflecting their increased translational validation and clinical utility. For this calculation, clinical trials from the last 6 years are included, which mitigates potential bias toward historical, well-validated targets with strong clinical precedent.

The resulting clinical target scores were subsequently used for training the ML models described below. We considered 3 distinct training scenarios:

• (All) all clinical targets: including all targets associated with any clinical trial phase
• (Approved) approved targets: restricted to those with a clinical phase value of 4 in ChEMBL for a given indication
• (Unique) indication-specific and shared targets: where only unique indication-specific targets or targets present across all indications were retained

Figure S1 summarizes the number of clinical targets used in each scenario, showing both the per-indication counts and the overlap between disease areas.

Note that the above scoring scheme was used only in the regression and multiclass classification experiments presented in the cross-validation comparison. In the binary classification setup—which yielded the best performance and was adopted for all subsequent target prediction and validation analyses—clinical scores are reduced to a binary label: any target with a positive clinical score is assigned the label 1, while negative samples receive the label 0. Under this binarization, the specific numerical values of the phase weights and the approval bonus have no influence on the training signal.

### Model training

We employed a supervised learning approach to train multiple ML models, using KG LP scores as input features and clinical scores as output labels. Because the clinical scores were continuous, we evaluated several modeling paradigms to explore both regression and classification performance:

• regression models: linear regression and *k*-neighbor regression
• multiclass classification models: random forest (RF) and support vector machine (SVM)
• binary classification models: RF and SVM

Prior to training, all clinical scores were log_2_-transformed to reduce skewness and stabilize variance. To improve model robustness and prevent overfitting, we augmented the dataset with negative samples, defined as genes not present among the clinical drug targets. These “negative targets” were randomly selected from the human genome excluding known drug targets and assigned a clinical score of −1. For multiclass classification models, continuous clinical scores were discretized into quantile-based bins (3, 4, and 6 bins) using the qcut() function from the pandas Python library. For binary classification models, positive clinical scores were considered ones and negative scores as zeros.

### San Antonio Breast Cancer Symposium targets

As an external validation set, we curated a list of emerging breast cancer targets from abstracts presented at the SABCS 2024 [30]. Abstracts were processed using a text-mining pipeline to identify sentences containing pharmacologically relevant keywords, including target(ing), inhibit(ing), degrade(r), agonist, directed, and bind(ing). Identified sentences were then screened for mentions of human protein-coding genes and their aliases, based on annotations obtained from the HGNC [33]. Candidate genes were cross-referenced against the set of known clinical breast cancer targets described above, and genes not present in this reference set were retained as putative novel therapeutic targets for breast cancer.

It is important to note that this external validation set was derived exclusively through text mining of the conference abstracts and therefore captures only the likely presence of a pharmacological drug–gene–disease association. As such, we do not distinguish between those that provide a positive effect on the disease or potential off-target or negative impacts. In addition, all candidate genes identified through this process were treated equivalently, without applying any weighting to reflect the strength, frequency, or experimental maturity of the supporting evidence. Therefore, from this gene set, we expect an enrichment in novel target genes, but that not all genes identified in this way to be potential drug targets for breast cancer.

#### Pathway genes

When working with SABCS to enhance the predictive performance and biological relevance of our models, we supplemented the training data for each indication with pathway-associated genes obtained using the Geneshot search engine [34]. The search terms used in pathway gene queries were as follows:

• cancer indications
  ◦ breast cancer (breast)
  ◦ lung cancer (lung)
  ◦ bowel cancer (bowel)
  ◦ prostate cancer (prostate)
  ◦ melanoma (melanoma)
• noncancer indications
  ◦ diabetes mellitus type 2 (diabetes)
  ◦ cardiovascular disease (cardiovascular)

These genes, identified based on literature co-occurrence and functional enrichment, were assigned a positive clinical score of 1 and included alongside the clinical drug targets during model training.

The returned pathway genes were sorted by their rank, and only the top *N* genes were used. Furthermore, only unique pathway genes—those not shared across multiple indications—and genes present in every indication were retained from the top *N* genes. This filtering step aimed to reduce redundancy and emphasize disease-specific molecular context.

During model training, the pathway genes are filtered by their intersection with the KG node set: only genes present as nodes in the KG (and thus possessing LP score features) are retained as additional positive training examples; genes absent from the KG are automatically excluded as they lack the embedding-derived feature vectors required for classification.

Given that many clinical targets share common properties across indications, we hypothesized that incorporating pathway genes would provide additional indication-level specificity and improve the model’s ability to distinguish between disease contexts.

In the results section, we consider 3 scenarios corresponding to obtaining the top 0, 100, and 300 pathway genes from the Geneshot search engine. These numbers change after the uniquification (Fig. S2); however, we still refer to them as 0, 100, and 300 in the text.

## Results

### Cross-validation

#### Number of KG nodes

Given that there is considerable variation in the size of the public domain KGs tested, we first assessed whether model performance was influenced by the size of the KG. To evaluate this, we conducted a cross-validation experiment using the Hetionet KG, the RotatE embedding model, and breast clinical targets. The KG feature space—defined as the number of node embeddings used as model input—was systematically downsampled to subsets containing 30,000, 20,000, 10,000, 5,000, 1,000, 500, 100, 50, 10, and 5 features. The corresponding performance metrics across these feature subsets are shown in Fig. S3.

Model performance remained largely stable across a broad range of feature sizes, with a notable decline only when the number of features dropped below approximately 1,000. To balance computational efficiency with predictive power for larger KGs, we therefore randomly selected 10,000 features for all KGs—one order of magnitude above the observed performance threshold—for LP and subsequent model training.

To verify that our results are not specific to a single embedding technique, we repeated the full cross-validation experiments using 3 additional embedding algorithms representing different relational modeling paradigms: TransE, ComplEx, and DistMult (see Methods). All 4 methods achieved strong binary classification performance across all KGs and indications, with the mean area under the receiver operating characteristic curve (AUROC) ranging from 0.870 (ComplEx) to 0.905 (DistMult), compared to 0.894 for RotatE (Fig. S4A). These results demonstrate that the downstream target prioritization is robust to the choice of embedding algorithm, and we therefore retain RotatE throughout the main analyses.

#### Performance

**Fig. 1.**
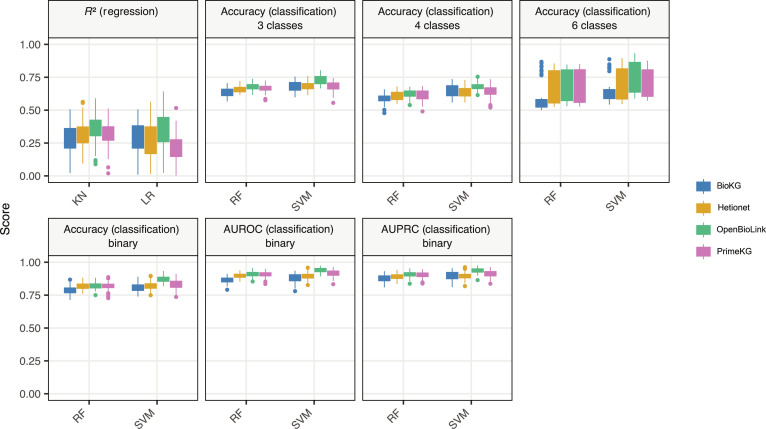
Cross-validation results of various regressors/classifiers. Each bar plot consists of 70 data points corresponding to 7 disease indications and 10 different random seeds for each indication. The baseline metrics for each indication are shown in Fig. S4B. KN, *k*-neighbor regression; LR, linear regression; RF, random forest; SVM, support vector machines; *R*^2^, coefficient of determination; AUROC, area under the receiver operating characteristic (ROC) curve; AUPRC, area under precision–recall curve.

To assess the predictive performance of the ML models, we conducted 5-fold cross-validation for each regressor and classifier (Fig. 1). For the multiclass classification experiments, we tested models with 2, 3, 4, and 6 discrete classes derived from the binned clinical target scores. We evaluated binary classifiers using AUROC, accuracy, and area under the precision–recall curve (AUPRC); the consistency between AUROC and AUPRC confirms that performance is not inflated by class imbalance.

Across all experiments, the RF and SVM models consistently achieved the highest predictive performance, particularly in the binary classification setup. Based on these results, subsequent analyses and target-ranking experiments in this study were performed using the RF model trained on binary (zero or nonzero) clinical scores.

To assess whether the LP score transformation provides an advantage over using raw entity embeddings directly, we repeated the cross-validation experiments using the trained embedding vectors as input features in place of LP scores. LP scores consistently outperformed raw embeddings across all evaluation settings (Fig. S4C). For binary classification, LP scores achieved a mean AUROC of 0.877 compared to 0.842 for raw embeddings and a mean AUPRC of 0.874 versus 0.845. Similar improvements were observed for multiclass classification (e.g., 3-class accuracy: 0.640 versus 0.603) and regression, where raw embeddings exhibited substantially higher variance. These results confirm that projecting entity embeddings into a relation-aware LP score space—capturing specific biological interaction types rather than abstract latent positions—yields more informative and stable features for downstream prediction, although it requires an extra computational analysis step.

To assess which relationship types contribute most to drug target predictions, we analyzed RF Gini feature importances aggregated by entity type for each KG. Raw total importance per type was dominated by the number of features in that category (Fig. S4D), so we computed the mean per-feature importance normalized against a uniform baseline (Fig. S4E). Across all 4 KGs, normalized importance was distributed relatively uniformly (range 0.85 to 1.27 of baseline), with no single relationship type dominating predictions. Modest enrichments were observed for pathway-related features (BioKG 1.05 and PrimeKG 1.27 ranges of baseline), anatomical features (Hetionet: 1.14 range of baseline), and Gene Ontology terms (OpenBioLink: 1.08 range of baseline). These results indicate that the predictive signal is spread across diverse biological relationship types.

### Model performance

**Fig. 2.**
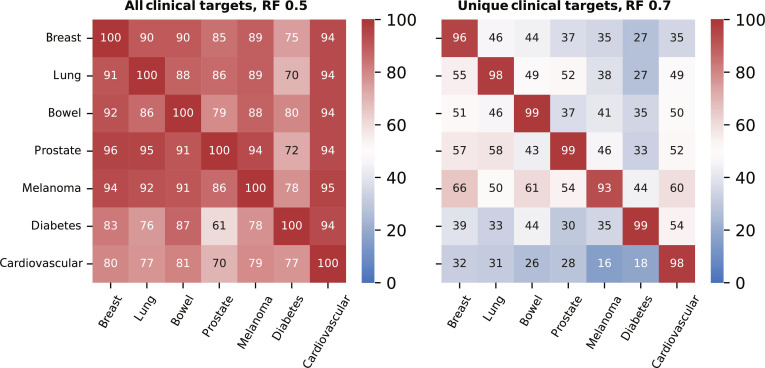
Heatmaps showing the percentages of common genes between predicted targets from *x*-axis indications and the input clinical targets (*y*-axis) for the Hetionet knowledge graph (KG). The left subfigure corresponds to models training on All clinical targets with a 0.5 random forest (RF) probability threshold. The right subfigure corresponds to models training on Unique clinical targets with a 0.7 RF probability threshold. Each cell is an average of 10 independent results corresponding to 10 different random seeds. A default RF probability threshold of 0.5 was used for model predictions. The results for the other KGs and other RF thresholds (0.6 to 0.9) are shown in Figs. S5 to S9.

To evaluate model performance, we assessed several metrics, focusing initially on specificity and sensitivity. These metrics were quantified by measuring the overlap between predicted targets and the corresponding clinical target sets for each disease indication. Overlaps were visualized as heatmaps, with disease indications represented on both axes (Fig. 2, Hetionet example). When models were trained using All clinical targets (see Methods) and evaluated with the default RF probability threshold (Fig. 2, left), the resulting heatmap was largely homogeneous, with values close to 100% across indications, showing limited specificity. In contrast, applying a stricter RF threshold and restricting the training set to Unique clinical targets (Methods, Fig. 2, right) substantially reduced off-diagonal overlap, while diagonal values remained close to 100%. This pattern indicates that the models retained the ability to recover their respective training targets while achieving improved indication specificity.

Based on these observations, we introduced the following custom metrics:

• Mean specificity was defined as 1 minus the average proportion of false positives across all other indications (i.e., 1 minus the mean of the off-diagonal values divided by 100).
• Mean sensitivity was defined as the proportion of true positives for each indication (i.e., the diagonal value divided by 100).

Intuitively, higher mean specificity corresponds to fewer off-target predictions (lower off-diagonal elements), while higher mean sensitivity indicates stronger recovery of known clinical targets (higher diagonal elements).

In addition to mean specificity and mean sensitivity, we introduced an additional performance metric to capture the overall frequency of positive predictions from the model. Specifically, the baseline predictivity was defined as the fraction of all genome genes that are predicted as targets for a given indication. This metric provides a global measure of model stringency, independent of any disease context. Baseline predictivity is particularly informative for target prioritization tasks, where the practical utility of a model depends not only on its ability to recover known targets but also on its capacity to discriminate novel targets such that positive predictions are obtained for a manageable and biologically meaningful subset of genes. A high baseline prediction level indicates a permissive model that nominates a large fraction of the genome as potential targets, reducing prioritization value and increasing the burden of subsequent validation. Conversely, a low baseline reflects a more selective model, yielding shorter, more actionable target lists suitable for downstream validation.

By jointly considering baseline predictivity alongside mean sensitivity and mean specificity, we ensure that model performance balances biological coverage, indication relevance, and practical usability in real-world drug discovery workflows.

### Parameter optimization

Our pipeline incorporated several tuneable parameters, including the probability threshold used for RF predictions, as well as the number of clinical targets included during model training. To identify the optimal parameter configuration, we performed a parameter optimization analysis using the 3 previously defined metrics: baseline, mean specificity, and mean sensitivity. Optimization was conducted across the following parameter space:

• clinical targets: All, Approved, and Unique (see Methods)
• RF probability thresholds: 0.5 (default), 0.6, 0.7, 0.8, and 0.9

**Fig. 3.**
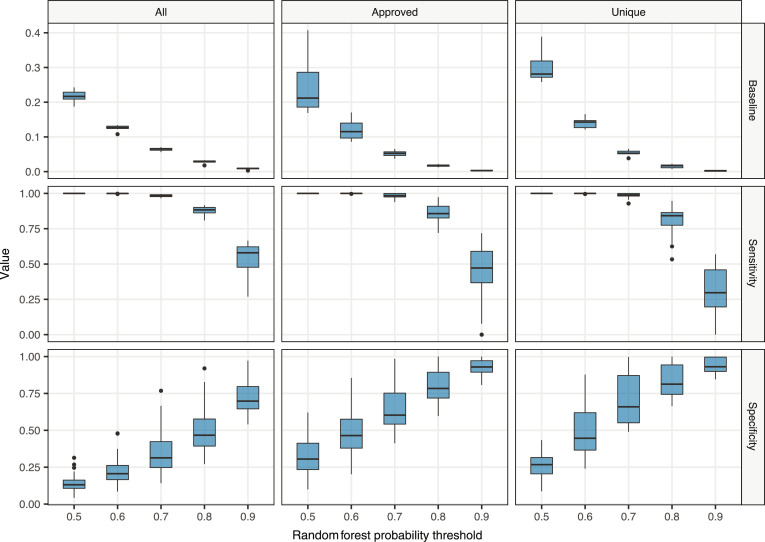
Optimization of mean specificity, mean sensitivity, and baseline metrics. Boxplots correspond to all knowledge graphs (KGs) and all disease indications.

The objective of the optimization was to jointly maximize mean specificity, mean sensitivity, and baseline performance. The analysis revealed several consistent trends (Fig. 3). As expected, mean specificity increased with more stringent filtering, both when progressively restricting the clinical target set (All → Approved → Unique) and when increasing the RF probability threshold (0.5 → 0.9). Mean sensitivity exhibited the opposite trend, although the decrease was comparatively modest. In contrast, baseline performance declined sharply with increasing RF probability thresholds but was consistently higher when the clinical target set was more restricted. Based on these trade-offs, the optimal parameter range was defined as

• using Unique clinical targets
• setting the RF probability threshold to 0.7

### San Antonio Breast Cancer Symposium targets

Having established the optimal parameter range, we next evaluated Artemis’s ability to identify drug targets beyond those already known from clinical data. As an external validation set, we used emerging novel breast cancer targets extracted from the SABCS 2024 abstracts (Methods).

**Fig. 4.**
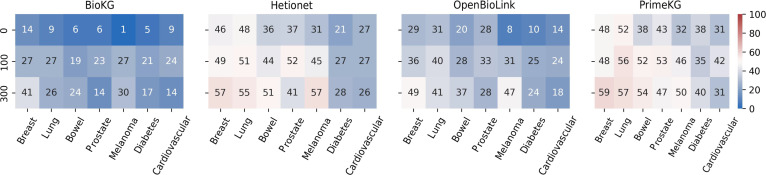
A heatmap showing percentages of common genes between predicted targets from *x*-axis indications and the San Antonio Breast Cancer Symposium (SABCS) novel targets. The *y*-axis represents the number of pathway genes used for training (Methods). Each cell is an average of 10 independent results corresponding to 10 different random seeds. The results for the non-default random forest (RF) thresholds are shown in Figs. S10 to S14.

In the same manner as in Fig. 2, we calculated the overlap between the predicted targets and the SABC targets for each indication and each KG (Fig. 4). Note that we expected that the text-mining approach to identify that the novel targets would contain a degree of inaccuracy in identifying gene names within an abstract to being a legitimate investigational drug target; therefore, 100% prediction would not be expected.

Although the breast cancer model was not uniformly the top-performing model, a clear separation emerged between cancer and noncancer indications. Models trained on noncancer diseases (diabetes and cardiovascular disease) showed minimal overlap with targets reported at SABCS, whereas models trained on cancer indications exhibited prediction patterns like the breast cancer model. This convergence suggests that shared oncogenic mechanisms are captured by the KG-derived features and contribute to cross-cancer target prioritization.

Incorporating pathway genes into the training set (Methods), using either 100 or 300 genes, consistently increased the number of predicted targets, indicating that pathway-level information provides complementary signal beyond clinically validated targets alone. This effect was substantially more pronounced for cancer indications than for noncancer diseases, consistent with the notion that cancers share common oncogenic pathways that may be discriminated by the added pathway context. Notably, in several settings, inclusion of pathway genes led to the strongest overall performance for the breast cancer model, showing the desired outcome.

Importantly, the positive classification rate for novel SABCS targets was substantially higher than the baseline prediction level (6% to 7%; Fig. 3), indicating that the models’ predictions are markedly enriched relative to random expectation.

**Fig. 5.**
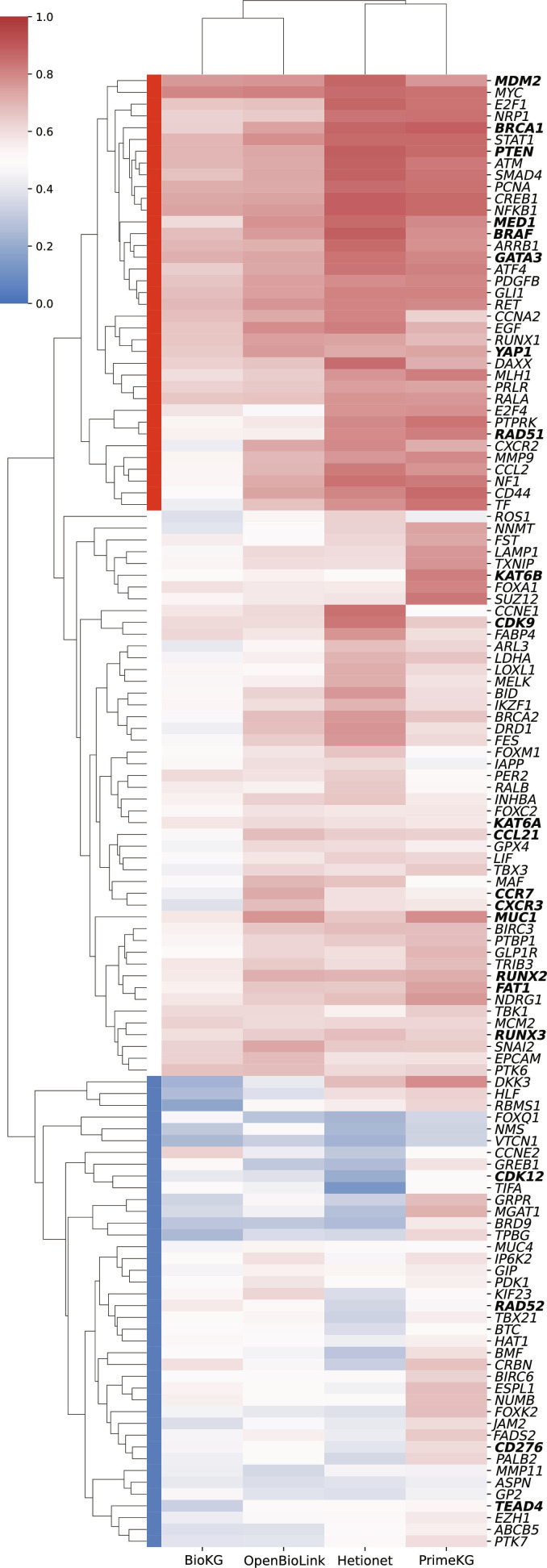
A heatmap of predicted random forest (RF) probabilities for San Antonio Breast Cancer Symposium (SABCS) novel breast cancer targets from a breast model without pathway genes (Methods). Cell values correspond to a mean across 10 runs with different random seeds. Only genes that were present in all 4 knowledge graphs (KGs) are present in the heatmap (genes that were not present in at least one KG were filtered out). Genes and KGs were clustered by hierarchical clustering that are shown as dendrograms. Three clusters described in the main text are highlighted with red/white/blue next to the dendrograms. Genes mentioned in the text are highlighted with bold font.

Furthermore, we compared the RF probability scores assigned to SABCS genes across different KGs (Fig. 5). Genes were grouped using hierarchical clustering, revealing 3 major clusters corresponding to high, intermediate, and low RF probability levels. Overall, the results indicate strong consistency in predictions across KGs. Where discrepancies between KGs were observed, primarily within the intermediate cluster, RF probabilities for ~60% to 70% of genes clustered around 0.5, suggesting uncertainty rather than systematic disagreement. In contrast, the remaining ~30% of genes showed larger divergences in RF probabilities, suggesting KG-specific effects.

The top-ranked predicted genes (red cluster) included several well-established players in breast cancer biology, many of which remain undrugged despite extensive evidence supporting their roles in tumorigenesis. These included *BRCA1*, *PTEN*, and *MED1* among others. Other targets in this bracket are those that have been explored for other oncology indications (e.g., *BRAF* and *MDM2*) and therefore could be considered potential areas for drug repurposing or for indication expansion. The analysis also selected genes such as *GATA3*, which is a differentiation marker of luminal breast cancer and associated with positive prognosis [35], indicating that activation of such a gene (or a related pathway) could be a therapeutic strategy. Notably, *YAP1*, a key mediator of the Hippo pathway that is frequently dysregulated in cancer, was also placed in this top cluster. *RAD51*, whose overexpression has been associated with resistance to PARP inhibitors, was similarly ranked among the top-predicted genes.

The mid-ranked portion of the predictions (white cluster) was particularly intriguing, as it highlighted divergence between KGs and may represent the most novel target space. This group included *FAT1*, a target of extensive current research due to its association with the emergence of resistance to CDK4/6 inhibitors [36] and its role in the Hippo/YAP pathway. Its presence in this cluster, alongside *YAP1* in the top-ranked cluster, substantiates the potential of the Hippo/YAP pathway as a source of therapeutic targets. *CDK9*, which has been explored for other oncology indications, was also placed in this intermediate bracket and could be considered for drug repurposing. MUC1 is a surface antigen across many cancers that has been found in a majority of triple-negative breast cancers and is being considered a target for antibody–drug conjugate and CAR-T therapies [37].

Also in this portion were both the chemokine receptor *CCR7* and the gene for its ligand *CCL21*. The *CCR7*/*CCL21* axis has been implicated in lymphogenesis and metastasis in breast cancer [38]. Similarly, *CXCR3*, a second chemokine receptor, is also associated with promoting metastasis in breast cancer [39]. These targets are indicative of emerging opportunities for the treatment of breast cancer through immunomodulation.

Other closely associated target clusters were also found in this set. *RUNX2* and *RUNX3* have been associated with aggressive, metastatic forms of breast cancer through their action in stabilizing MYC [40]. The genes encoding lysine acetyltransferase enzymes *KAT6A* and *KAT6B*, both found in this cluster, are epigenetic modulators and indicated as potential novel targets in numerous cancers settings but may be particularly relevant to breast cancer due to their high frequency of genomic alterations in patients [41].

The lower-ranked genes (blue cluster) presented a more heterogeneous mix. Some, such as *CDK12* and *RAD52*, are likely false negatives, as they are well established in DNA repair pathways and PARP inhibitor resistance. *TEAD4*, a downstream effector of the Hippo/YAP pathway, was also placed in this cluster, further illustrating the variable ranking of Hippo pathway components across KGs. *CD276* (B7-H3) encodes a surface antigen that has been reported to be overexpressed in many cancers, but antibodies and CAR-T therapeutics against this target have had limited clinical success to date. The remaining genes are less characterized, representing potentially novel candidates for further biological validation.

## Discussion

In this work, we introduced a practical framework, Artemis, that harnesses several publicly available KGs to systematically prioritize therapeutic targets for defined disease indications. The approach aggregates network-derived characteristics of already established drug targets, uses these features to train an ML model, and then applies the model to score previously uncharacterized candidates. Importantly, the training set is anchored in targets supported by clinical evidence of efficacy within the relevant disease context. We demonstrated that this methodology is broadly applicable by constructing models for a range of cancer and noncancer indications and observing consistently strong predictive performance.

**Fig. 6.**
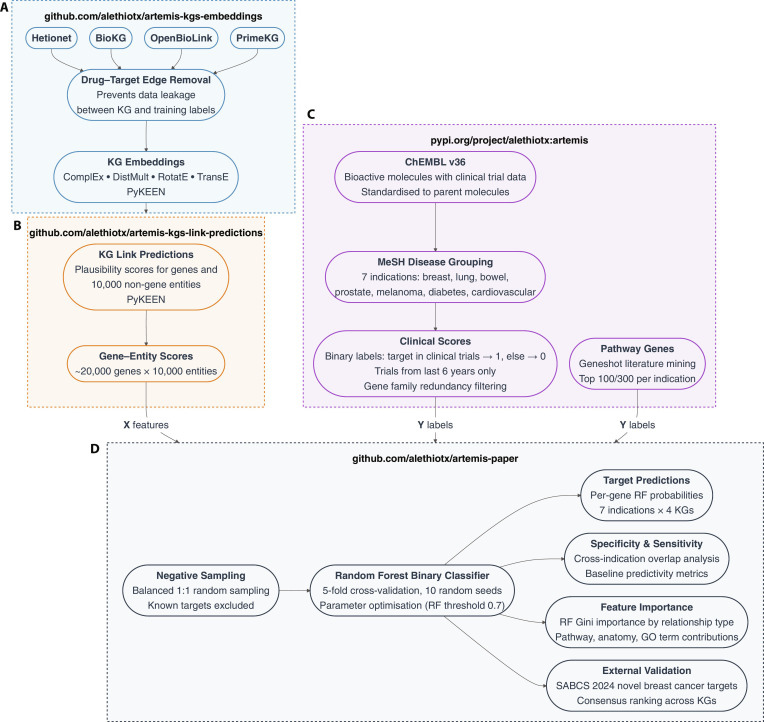
Overview of the Artemis framework for indication-aware target prioritization. Dashed borders denote independent pipeline components. (A) Four public knowledge graphs (KGs) are preprocessed with drug–target edge removal and embedded using ComplEx, DistMult, RotatE, and TransE via PyKEEN. (B) Link-prediction scores for every gene paired with 10,000 nongene entities yield a feature matrix. (C) Clinical labels are derived from ChEMBL v36 trial data grouped into 7 indications via Medical Subject Headings (MeSH), supplemented by pathway genes from Geneshot. (D) A random forest classifier trained on balanced datasets and tested under 5-fold cross-validation produces per-gene target probabilities across all KG–indication combinations.

Figure 6 summarizes the end-to-end framework and highlights 3 design choices that distinguish it from existing KG-based target identification approaches. First, the systematic combination of 4 public KGs with 4 embedding algorithms provides robustness against structural biases inherent in any single graph, allowing consensus predictions that are not artifacts of a particular KG’s curation scope. Second, the use of LP plausibility scores as features, encoding each gene’s topological relationships to biomedical entities, eliminates the need for manual feature engineering and captures biological context that single-relation approaches miss. Third, the integration of independently derived clinical labels from ChEMBL trial data and literature-mined pathway genes grounds predictions in disease-specific evidence, addressing a limitation of purely topology-driven methods.

The clinical score derivation and pathway gene retrieval components are distributed as a standalone Python package (alethiotx:artemis, available on PyPI), decoupling label construction from the downstream modeling pipeline. This allows users to generate up-to-date indication-specific training labels, querying ChEMBL for trial-stage targets and Geneshot for literature-mined pathway genes, independently of the KGE or classification stages. By packaging this functionality separately, label sets can be regenerated on demand as ChEMBL releases new versions or as disease ontologies evolve, without requiring changes to the rest of the framework.

Critically, the entire framework is implemented as a set of containerized, orchestrated modules (Nextflow pipelines [42]), enabling fully automated re-execution without manual intervention. As upstream KGs release new versions or ChEMBL incorporates additional trial outcomes, the pipeline can be rerun end to end to produce updated target rankings. The modular architecture further allows individual components, KG sources, embedding methods, label definitions, or classifiers, to be substituted or extended independently, supporting both methodological iteration and adaptation to new therapeutic areas beyond the 7 indications demonstrated here.

Beyond the indications studied here, the Artemis framework can be extended along several axes. Most directly, it can be applied to any new disease indication for which clinical trial data exist in ChEMBL: the pipeline automatically constructs indication-specific training labels via MeSH disease grouping and trains dedicated models without manual curation. We demonstrated this across 7 diverse indications spanning 5 cancers, diabetes, and cardiovascular disease, but the same mechanism generalizes to neurodegenerative, autoimmune, or infectious disease contexts given sufficient trial coverage. The modular architecture also accommodates alternative or additional KGs—any KG provided in standard triple format can be embedded and integrated without modifying the downstream classification pipeline, enabling users to incorporate domain-specific or newly published graphs as they become available. Looking beyond target identification, the framework naturally lends itself to adjacent applications. For drug repurposing, genes predicted as high-probability targets for one indication can be cross-referenced against existing drug–target mappings to generate systematic repositioning hypotheses. For biomarker discovery, the feature importance structure of the RF models, decomposed by relationship type and entity class, can highlight which biological relationships are most predictive of clinical relevance, potentially revealing novel biomarker candidates. Finally, because the pipeline is fully automated, it can be re-executed as ChEMBL and public KGs are updated, enabling temporal monitoring of how target prioritization landscapes evolve and providing a living resource for drug discovery teams.

To clarify the positioning of Artemis relative to existing approaches, we provide a structured comparison in Table 3. Broadly, existing methods fall into 3 categories: (a) KG-centric LP systems [9–21] that use graph embeddings or structure as the direct predictive output, (b) evidence aggregation platforms such as Open Targets that score target–disease associations by integrating heterogeneous preexisting data sources without performing predictive modeling, and (c) proprietary multisignal frameworks such as the AstraZeneca BIKG-based recommendation system [43] that combine KG features with additional ranking signals but depend on inaccessible infrastructure. Artemis is architecturally distinct from all 3: it employs KG LP scores as input features to a supervised classifier trained on indication-specific clinical labels from ChEMBL, enabling the model to learn which network signatures characterize clinically validated targets for a given disease. This 2-stage design allows ranking of novel targets that lack prior direct clinical evidence, a capability that evidence aggregation platforms do not possess, while grounding predictions in empirical translational data rather than graph topology alone.

**Table 3.** Comparison of Artemis with existing target discovery approaches

Feature	KG-centric methods	Open Targets	AZ BIKG framework	Artemis
Primary approach	KG LP/embedding-based scoring	Evidence aggregation	Proprietary KG + multisignal optimization	KG features → supervised ML on clinical labels
Role of KG	Final predictive output	One of many evidence sources	Feature source + proprietary construction	Input feature space for downstream classifier
Indication-specific models	Generally no	Scores per disease (no ML)	Single indication	Seven indications demonstrated
Clinical evidence integration	None or minimal	Aggregates existing annotations	Limited	ChEMBL clinical scores as training labels
Infrastructure	Varies	Open platform	Proprietary	Fully open (public KGs + open-source code)
Reproducibility	Partial	N/A (platform)	Not reproducible externally	Fully reproducible (Nextflow + public data)

KG, knowledge graph; AZ BIKG, AstraZeneca Biological Insights Knowledge Graph; LP, link prediction; ML, machine learning; N/A not applicable

We assessed Artemis’s performance using cross-validation and introduced quantitative metrics to guide parameter optimization. This procedure identified a binary RF model with a probability threshold of 0.7 as the best-performing configuration.

The selection of binary classification as the primary evaluation framework was motivated by 2 complementary considerations. First, binary classification consistently yielded the strongest cross-validation performance across KGs and disease indications (Fig. 1), which we interpret as evidence that the binary formulation best captures the underlying signal structure in the LP score features. Second, binary classification is the most natural formulation for the downstream task of preclinical target prioritization, where the actionable question is inherently dichotomous: whether a gene warrants further investigation as a therapeutic target for a given indication. While the continuous clinical score (regression) and binned categories (multiclass) encode useful gradations of clinical advancement, these gradations do not directly map to the decision framework employed in drug discovery, where teams require a ranked list of candidates above a confidence threshold. The practical validity of this choice is further supported by the SABCS 2024 external validation, in which the binary classification model successfully identified genes under active translational investigation, confirming that the binary formulation provides sufficient granularity for real-world target discovery.

One potential limitation of our approach is the reliance on random negative sampling from unlabeled genes, which may include undiscovered therapeutic targets misclassified as negatives. To assess the impact of this, we conducted 2 supplementary analyses. First, we measured prediction stability across the 10 training iterations, each using an independently drawn set of negatives: the cross-validation AUROC standard deviation was low (mean 0.016, max 0.047), the mean pairwise Jaccard similarity of predicted target sets was 0.71, and 55% of predicted genes were consistently identified in at least 8 out of 10 iterations (Figs. S15 to S17). Second, we implemented a confident-negative selection procedure in which an initial model was used to score all unlabeled genes and only those with a low predicted target probability were retained as negatives for retraining (Figs. S18 to S20). The gene-level probability rankings between the standard and confident-negative models were highly correlated (mean Pearson *r* = 0.88), indicating that the relative ordering of candidate targets is preserved regardless of the negative sampling strategy. We also considered positive-unlabeled learning as an alternative; however, positive-unlabeled methods assume that labeled positives are selected completely at random from the true positive set, whereas clinical trial targets are systematically biased toward well-characterized druggable gene families, violating this assumption. Together, these results suggest that our conclusions are robust to the choice of negative samples.

When the Artemis platform was applied to the set of genes identified from the SABCS 2024 abstracts (Methods), all KGs showed positive predictions for breast cancer substantially higher than the genome-wide baseline level. Cancer indications achieved comparable target recovery rates across all KGs and pathway gene configurations (Fig. 4), indicating that the framework captures shared oncogenic biology—consistent with the established hallmarks of cancer, wherein PI3K/AKT, RAS/MAPK, and cell cycle pathways drive tumorigenesis across tissue types, and with the clinical reality that many targeted therapies are evaluated across multiple tumor types simultaneously. Among cancers, prostate showed the lowest recovery, possibly reflecting its distinct hormonal biology. Diabetes and cardiovascular disease consistently showed lower positive prediction rates for the SABCS targets, although still above baseline levels; this clear separation between oncology and non-oncology indications confirms that the model does not merely learn generic “druggability” features but captures indication-relevant biology. The relatively uniform performance across cancer subtypes suggests that high-confidence predictions within oncology are likely enriched for pan-cancer targets, and users seeking tumor-type-specific candidates may benefit from post hoc filtering strategies such as tissue-specific expression or indication-specific pathway constraints. Adding pathway genes to the training set had a pronounced impact on the prediction of novel SABCS candidates (Fig. 4), underscoring the value of pathway-derived features for identifying previously uncharacterized disease-relevant genes.

Examination of the highest-ranked SABCS predictions revealed several well-established genes in breast cancer biology, many of which remain undrugged despite extensive evidence supporting their relevance. At the opposite end of the ranking, the weakest predictions were largely genes with limited or no known involvement in breast cancer, although a small number were likely false positives. The mid-ranked group was particularly noteworthy, as it contained genes that may represent genuinely novel and emerging target candidates.

These observations suggest that relying solely on KG-derived features even with the clinical data may still be insufficient for fully resolving target novelty or prioritization. Incorporating additional, orthogonal ranking sources from publicly available resources could further refine prediction quality. For example, the AstraZeneca BIKG-based recommendation system [43] integrates multiple independent ranking signals and applies an optimization algorithm to identify the most promising targets, a strategy that could complement and enhance Artemis’s prioritization pipeline.

This work illustrates the value of applying clinically grounded, KG–driven ML to therapeutic target prioritization. This is particularly useful where modern genomic approaches, such as CRISPR screening, single-cell expression analysis, and sequencing of patient sample banks, generate lists of 10s to 100s of genes as potential therapeutic targets. While the analyses presented here focus on selected cancer and noncancer indications, the underlying methodology is disease agnostic and makes Artemis readily transferable to other disease areas where there is a critical unmet need for novel therapeutic targets.

## Data Availability

All external and generated data used in this study are publicly available in the Amazon Web Services (AWS) S3 bucket (s3://alethiotx-artemis), which can also be browsed at http://alethiotx-artemis.s3-website.eu-west-2.amazonaws.com/. The core analyses are implemented in Python and provided in the artemis module of the alethiotx Python package (https://pypi.org/project/alethiotx/), with full documentation available at https://alethiotx.github.io/pypi/alethiotx/artemis.html. A fully reproducible Nextflow [42] pipeline for generating all data and figures in this article is available at https://github.com/alethiotx/artemis-paper. Additional Nextflow pipelines for KGE generation and KG LP are available at https://github.com/alethiotx/artemis-kgs-embeddings and https://github.com/alethiotx/artemis-kgs-link-predictions, respectively. All workflows were executed on the Seqera Platform (https://cloud.seqera.io/) using AWS Batch and read from and write to the same S3 bucket; embedding generation leveraged g5 instances with NVIDIA A10G Tensor Core graphics processing units.

## Supplementary Materials

Supplementary 1 Supplementary Notebook Figs. S1 to S20
