# Supplementary material for: Artemis: Harnessing Knowledge Graphs for Next-Generation Drug Target Prioritization
Source: Comput Struct Biotechnol J. 2026 Jun 26;35(1):0130. doi: 10.34133/csbj.0130 (PMC13484285; doi:10.34133/csbj.0130)
Supplement: Supplementary file 1 [file csbj.0130.f1.zip › Supplementary Figures_revised.docx]

Figure 1. Upset plots ([https://upsetplot.readthedocs.io](https://upsetplot.readthedocs.io/)) showing the number of clinical targets (obtained on 2025-12-12) used in model training after selecting either: 1. All the targets (top subplot), not showing intersections with the size smaller than 10; 2. Only FDA approved targets (bottom left subplot), not showing intersections with the size smaller than 10; 3. Either unique indication-specific targets or targets present in all indications (bottom right subplot).

Figure 2. Upset plots ([https://upsetplot.readthedocs.io](https://upsetplot.readthedocs.io/)) showing the number of pathway genes (obtained on 2025-12-12) used in training after obtaining the top 100 (left) and 300 (right) pathway genes for each indication via Geneshot and selecting only the unique indication-specific genes and the genes that are present in all indications.

Figure 3. Top - cross-validation (5-fold) results of the random forest classifier based on the Hetionet KG, RotatE embedding model and binary breast cancer targets. Bottom – same as the top figure but with the randomly shuffled training labels. For each point on the x axis KG features were randomly selected 10 times. Solid line corresponds to the polynomial fit with 7 degrees. Black vertical line corresponds to the threshold of 10,000 random features that was used in all KGs to select features for training.

**A**

**B**

**С**

**D**

**E**

Figure 4. Cross-validation results of various regressors/classifiers. **A)** The plot corresponds to real training targets; **B)** plot corresponds to baseline performance when training target scores are randomly shuffled. Each bar plot consists of 10 data points corresponding to 10 different random seeds for each indication; **С)** Cross-validation performance comparison between link prediction (LP) scores and raw entity embeddings; **D)** Raw Gini importance mirrors feature count proportions. Comparison of the fraction of features belonging to each relationship type (blue) versus the fraction of total Gini importance attributed to that type (red). The near-identical values demonstrate that unnormalized importance is dominated by the number of features per type, motivating the per-feature normalization used in **E**; **E)** Per-feature importance of relationship types across knowledge graphs. Random Forest Gini importance was computed for each feature and averaged within relationship types, then normalized relative to a uniform baseline (dashed red line at 1.0). Values above 1.0 indicate relationship types that carry more predictive signal per feature than expected by chance. Results are aggregated across 7 disease indications (breast, lung, bowel, prostate cancer, melanoma, type 2 diabetes, cardiovascular disease), 3 random seeds, and the RotatE embedding. Each panel shows only the entity types native to that knowledge graph. KN – k-neighbour regression, LR – linear regression, RF – random forest, SVM – support vector machines. R^2 - coefficient of determination, ARI – adjusted Rand index, AUROC – area under the ROC curve, AUPRC – area under the precision-recall curve.

Figure 5. A heatmap showing percentages of common genes between predicted targets from x-axis indications and the input clinical targets (y axis). Each cell is an average of 10 independent results corresponding to 10 different random seeds. No pathway genes were used in training. RF probability threshold of 0.5 was used for model predictions.

Figure 6. A heatmap showing percentages of common genes between predicted targets from x-axis indications and the input clinical targets (y axis). Each cell is an average of 10 independent results corresponding to 10 different random seeds. No pathway genes were used in training. RF probability threshold of 0.6 was used for model predictions.

Figure 7. A heatmap showing percentages of common genes between predicted targets from x-axis indications and the input clinical targets (y axis). Each cell is an average of 10 independent results corresponding to 10 different random seeds. No pathway genes were used in training. RF probability threshold of 0.7 was used for model predictions.

Figure 8. A heatmap showing percentages of common genes between predicted targets from x-axis indications and the input clinical targets (y axis). Each cell is an average of 10 independent results corresponding to 10 different random seeds. No pathway genes were used in training. RF probability threshold of 0.8 was used for model predictions.

Figure 9. A heatmap showing percentages of common genes between predicted targets from x-axis indications and the input clinical targets (y axis). Each cell is an average of 10 independent results corresponding to 10 different random seeds. No pathway genes were used in training. RF probability threshold of 0.9 was used for model predictions.

Figure 10. A heatmap (based on 0.5 RF probability threshold) showing percentage of common genes between predicted targets from x-axis indications and the SABCS novel targets. Y axis represents the number of pathway genes used for training (Methods). Each cell is an average of 10 independent results corresponding to 10 different random seeds.

Figure 11. A heatmap (based on 0.6 RF probability threshold) showing percentage of common genes between predicted targets from x-axis indications and the SABCS novel targets. Y axis represents the number of pathway genes used for training (Methods). Each cell is an average of 10 independent results corresponding to 10 different random seeds.

Figure 12. A heatmap (based on 0.7 RF probability threshold) showing percentage of common genes between predicted targets from x-axis indications and the SABCS novel targets. Y axis represents the number of pathway genes used for training (Methods). Each cell is an average of 10 independent results corresponding to 10 different random seeds.

Figure 13. A heatmap (based on 0.8 RF probability threshold) showing percentage of common genes between predicted targets from x-axis indications and the SABCS novel targets. Y axis represents the number of pathway genes used for training (Methods). Each cell is an average of 10 independent results corresponding to 10 different random seeds.

Figure 14. A heatmap (based on 0.9 RF probability threshold) showing percentage of common genes between predicted targets from x-axis indications and the SABCS novel targets. Y axis represents the number of pathway genes used for training (Methods). Each cell is an average of 10 independent results corresponding to 10 different random seeds.

Figure 15. Cross-validation AUROC stability across negative sampling iterations. Mean AUROC (± standard deviation) for the RF classifier across 10 training iterations, each using an independently drawn set of negative samples. Results are shown for all combinations of knowledge graphs (BioKG, Hetionet, OpenBioLink, PrimeKG) and embedding methods (ComplEx, DistMult, RotatE, TransE), stratified by disease indication. The low variance (mean SD = 0.016) demonstrates that model discrimination is robust to the specific choice of negative samples.

Figure 16. Per-gene prediction stability across negative sampling iterations. (Top) Mean prediction stability, defined as the fraction of 10 iterations in which each gene is predicted as a target (RF probability ≥ 0.5), averaged across all genes predicted at least once. Values are shown per disease indication and KG-embedding combination. (Bottom) Number of genes consistently predicted as targets in at least 8 out of 10 iterations (≥80% stability), indicating the size of the robust core target set for each configuration.

Figure 17. Pairwise Jaccard similarity of predicted target sets across negative sampling iterations. For each KG-embedding-indication combination, target sets from all 10 iterations were compared pairwise, and the mean Jaccard index is reported. Higher values indicate greater consistency in the predicted targets regardless of which specific genes were sampled as negatives. The overall mean Jaccard similarity was 0.71 (range 0.53-0.83).

Figure 18. Correlation of predicted target probabilities between standard and confident-negative models. For each KG-embedding-indication combination, a heatmap shows the mean Pearson correlation coefficient between gene-level probability scores from the standard model (trained with balanced random negatives) and the confident-negative model (retrained using only low-probability unlabeled genes as negatives). The high mean correlation (r = 0.88) indicates that both approaches rank genes similarly.

Figure 19. Comparison of target sets predicted by standard and confident-negative models. (Top) Mean Jaccard index per KG–embedding–indication combination across 10 iterations. The Jaccard index is defined as J(A,B)=∣A∩B∣/∣A∪B∣. The moderate values (mean 0.43) reflect a difference in set size rather than disagreement. (Bottom) Recovery rate: fraction of standard-model targets also predicted by the confident-negative model. Values range from 0.986 to 1.000, confirming that the confident model subsumes nearly all standard predictions while predicting a larger overall target set that inflates ∣A∪B∣ (see Supplementary Figure 20).

Figure 20. Comparison of predicted target counts between standard and confident-negative models. Each point represents one KG-embedding-indication-iteration combination. The diagonal reference line (red dashed) indicates equal target counts. Points above the line show that the confident-negative model consistently predicts more targets (mean 10,635 vs. 4,531), as filtering to high-confidence negatives produces a more permissive classifier that subsumes the standard predictions.
